# Role of Glycogen Synthase Kinase-3 in Interferon-γ-Mediated Immune Hepatitis

**DOI:** 10.3390/ijms23094669

**Published:** 2022-04-23

**Authors:** Chia-Ling Chen, Po-Chun Tseng, Rahmat Dani Satria, Thi Thuy Nguyen, Cheng-Chieh Tsai, Chiou-Feng Lin

**Affiliations:** 1 School of Respiratory Therapy, College of Medicine, Taipei Medical University, Taipei 110, Taiwan; chialing66@tmu.edu.tw; 2 Core Laboratory of Immune Monitoring, Office of Research & Development, Taipei Medical University, Taipei 110, Taiwan; pctseng@tmu.edu.tw; 3 International Ph.D. Program in Medicine, College of Medicine, Taipei Medical University, Taipei 110, Taiwan; dr.dani.satria@gmail.com (R.D.S.); ntthuy.ub@huemed-univ.edu.vn (T.T.N.); 4 Department of Clinical Pathology and Laboratory Medicine, Faculty of Medicine, Public Health and Nursing, Universitas Gadjah Mada, Yogyakarta 55281, Indonesia; 5 Clinical Laboratory Installation, Dr. Sardjito Central General Hospital, Yogyakarta 55281, Indonesia; 6 Department of Microbiology and Immunology, School of Medicine, College of Medicine, Taipei Medical University, Taipei 110, Taiwan; 7 Department of Oncology, Hue University of Medicine and Pharmacy, Hue University, Hue City 530000, Vietnam; 8 Department of Nursing, Chung Hwa University of Medical Technology, Tainan 703, Taiwan; 9 Department of Long Term Care Management, Chung Hwa University of Medical Technology, Tainan 703, Taiwan; 10 Graduate Institute of Medical Sciences, College of Medicine, Taipei Medical University, Taipei 110, Taiwan

**Keywords:** glycogen synthase kinase-3, immune hepatitis, interferon-γ, liver

## Abstract

Glycogen synthase kinase-3 (GSK-3), a serine/threonine kinase, is a vital glycogen synthase regulator controlling glycogen synthesis, glucose metabolism, and insulin signaling. GSK-3 is widely expressed in different types of cells, and its abundant roles in cellular bioregulation have been speculated. Abnormal GSK-3 activation and inactivation may affect its original bioactivity. Moreover, active and inactive GSK-3 can regulate several cytosolic factors and modulate their diverse cellular functional roles. Studies in experimental liver disease models have illustrated the possible pathological role of GSK-3 in facilitating acute hepatic injury. Pharmacologically targeting GSK-3 is therefore suggested as a therapeutic strategy for liver protection. Furthermore, while the signaling transduction of GSK-3 facilitates proinflammatory interferon (IFN)-γ in vitro and in vivo, the blockade of GSK-3 can be protective, as shown by an IFN-γ-induced immune hepatitis model. In this study, we explored the possible regulation of GSK-3 and the potential relevance of GSK-3 blockade in IFN-γ-mediated immune hepatitis.

## 1. Multiple Roles of Glycogen Synthase Kinase-3 (GSK-3) in Human Diseases

**Figure 1:**
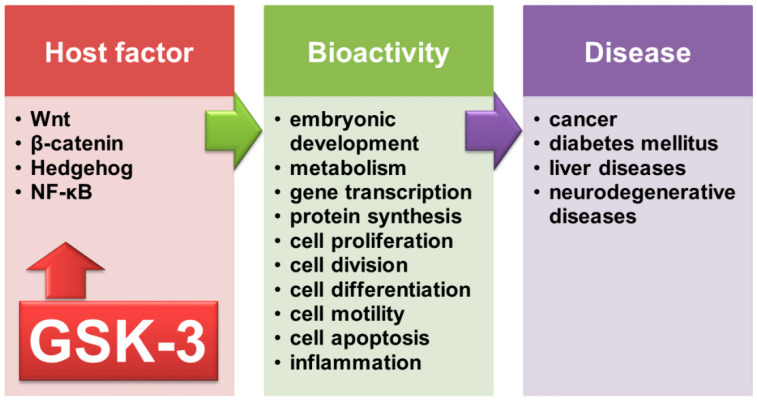
The various roles of GSK-3 contribute to diverse bioactivities and human diseases.

Glycogen synthase kinase-3 (GSK-3) was first recognized as a critical glycogen synthase and glycogen regulator responding to insulin signaling and glucose metabolism [1]. With regard to glycogen being made and stored primarily in the liver, particularly in hepatocytes, controlling glycogen by glycogen synthase is essential. GSK-3 consists of GSK-3α and GSK-3β [2] and is primarily expressed in the cytosol and nucleus in response to stimuli [3]. In response to growth factor withdrawal and starvation, GSK-3 is activated and then phosphorylates glycogen synthase to deactivate its enzymatic activity. In contrast, in response to blood glucose, insulin, and insulin-like growth factor (IGF) 1, GSK-3 is generally inactivated, and glycogen synthase is next activated to process glycogen biosynthesis. In addition, nuclear GSK-3 facilitates the phosphorylation of nuclear cyclin D1 in the S phase of the cell cycle [4]. However, GSK-3α and GSK-3β have different biological roles; the induction of embryonic lethality has been shown in GSK-3β but not GSK-3α knockout mice [5]. In an early study, GSK-3 was also found to participate in various biological processes by modulating Wnt/β-catenin, Hedgehog, and nuclear factor κB (NF-κB) signaling [6]. The multifactorial actions of GSK-3 are exhibited by its multiple intracellular substrates involving signaling, structure, and transcription [7] and regulate several cellular processes, including embryonic development, metabolism, gene transcription, protein synthesis, cell proliferation and division, differentiation, motility, apoptosis, and inflammation [1,8]. Hence, aberrant activation and inactivation of GSK-3 have been implicated in cancer, diabetes mellitus, liver diseases, and neurodegenerative diseases [9,10]. As an important regulator in response to diverse stimuli, the possible roles of GSK-3 are therefore summarized in Figure 1.

## 2. Regulation of GSK-3 in Facilitating Proapoptosis and Proinflammation

**Figure 2:**
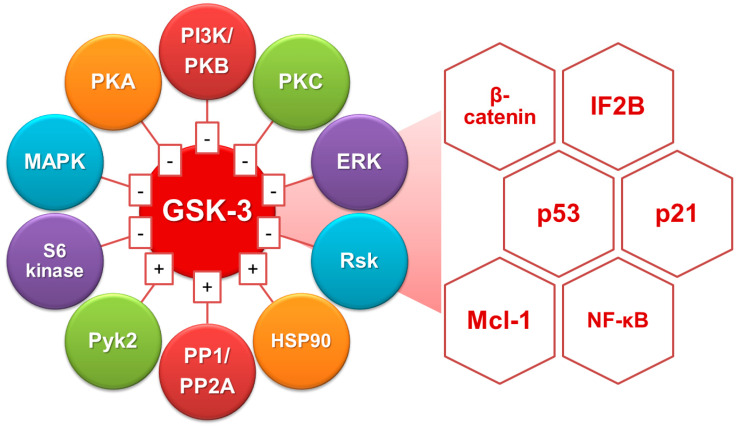
Molecular regulation of GSK-3 for activating the diverse intracellular factors.

Regulation of GSK-3 activation is suggested to be necessary for controlling many vital intracellular factors (Figure 2). First, GSK-3 inhibition by phosphorylation is regulated at the N-terminal serine 9 residue through phosphatidylinositol 3-kinase (PI3K)-Akt (protein kinase B, PKB) [11]. Pharmacological blockade of PI3K-Akt signaling causes GSK-3β dephosphorylation and activation followed by cell apoptosis in a GSK-3β-regulated manner [12,13]. Furthermore, activation of protein phosphatases, including protein phosphatase (PP) 1 and PP2A, can directly or indirectly dephosphorylate GSK-3β for activation by causing Akt dephosphorylation [14]. Additionally, the signaling pathways of the extracellular signal-regulated kinase (ERK), PKA, PKC, mitogen-activated protein kinase (MAPK)-activated protein kinase-1 (also known as p90rsk), p70 ribosomal S6 kinase, and Wnt activation also promote GSK-3 inactivation [7]. Alternatively, tyrosine kinases such as proline-rich tyrosine kinase (Pyk) 2 [15], MAPK/ERK kinase, and Src-like kinase regulate GSK-3 activity [7]. Moreover, a heat shock protein 90-mediated autophosphorylation mechanism has been suggested as a regulatory factor [16].

The proapoptotic role of GSK-3 is suggested in Alzheimer’s disease [17]. GSK-3 overexpression in target cells induces apoptosis [13,18]. Therefore, GSK-3 activation has been reported in apoptotic stimuli, including endoplasmic reticulum (ER) stress, growth factor withdrawal, heat shock, hypoxia, staurosporine administration, and mitochondrial complex I inhibition [12,18,19,20]. GSK-3 exerts its multiple regulatory actions on apoptosis through different mechanisms. Interactions of GSK-3β with β-catenin, initiation factor 2B, p21^Cip1^, and p53 translation may modulate cell fate in survival and apoptosis [7,13]. The current study demonstrated the novel proapoptotic role of GSK-3 by negatively regulating myeloid cell leukemia (Mcl)-1 protein followed by triggering mitochondrial damage [21]. PP2A and PI3K-Akt modulate GSK-3β activity, and GSK-3β, in turn, regulates mitochondrial permeability in ceramide-induced apoptosis [22]. In response to the ER stressor tunicamycin, GSK-3 is essential for cell apoptosis [23]. These molecular regulations show the proapoptotic role of GSK-3.

Disrupting the *GSK-3β* gene causes embryonic lethality [5]. In GSK-3β-deficient mice, severe liver degeneration results from excessive tumor necrosis factor-α (TNF-α) cytotoxicity. Significantly, GSK-3β can affect the early stage of NF-κB activation by interfering with cytosolic IκB degradation and nuclear translocation of NF-κB. The data indicate that GSK-3β regulates NF-κB signaling at the transcriptional complex. The potential regulation of NF-κB activation by GSK-3 was demonstrated in lipopolysaccharide (LPS)/Toll-like receptor (TLR)-4 and TNF-α/TNF receptor signaling. Further studies demonstrated that inhibiting GSK-3β protects cells from inflammatory stimuli, including endotoxemia [24], experimental autoimmune encephalomyelitis [25], experimental colitis [26], TNF-α [27], type II collagen-induced arthritis [28], TLR-mediated inflammatory responses [29,30], and OVA-induced asthma. Furthermore, GSK-3 regulates the expression of nitric oxide (NO), inducible NO synthase (iNOS), and regulated on activation, normal T-cell expressed and secreted (RANTES) in LPS-activated macrophages and endotoxemia-induced acute renal failure [31,32]. Furthermore, inhibiting GSK-3 results in an anti-inflammatory effect in LPS/interferon (IFN)-γ- and heat-inactivated staphylococcal aureus-activated macrophages and microglia [33,34]. A study on the therapeutic mechanisms of GSK-3 inhibition will help to understand the proinflammatory role of GSK-3. Because the activation of NF-κB is involved in various immune responses, GSK-3 is speculated to be proinflammatory and could be a therapeutic target for anti-inflammation [5,24,31,32].

## 3. Targeting GSK-3 as a Protective Strategy against Hepatic Injury

The therapeutic effects of GSK-3 blockade on hepatic protection have been demonstrated in TLR-mediated systemic inflammation involving multiorgan failure, including the lungs, liver, pancreatic injury, and renal dysfunction [35]. In diseased mice with GSK-3 inhibitor treatment, proinflammatory and proapoptotic molecules such as iNOS, nitrotyrosine, poly(ADP-ribose), CD30, CD30 ligand, and Fas ligand are markedly reduced. In a murine model of liver partial warm ischemia/reperfusion injury (IRI), active GSK-3 favors the development of liver pathology, while GSK-3 inhibitor ameliorates the hepatocellular injury as indicated by the presence of aspartate aminotransferase and histopathological examination [36]. Therefore, the findings on the pathogenic role of active GSK-3 are essential for explaining how carbon monoxide works to protect the IRI liver [37]. Carbon monoxide treatment causes activation of PI3K-Akt signaling to deactivate GSK-3. Notably, in these diseased mice with GSK-3 inhibitor treatment [36], the induction of anti-inflammatory interleukin (IL)-10 is essential for liver protection while neutralizing IL-10 overcomes the therapeutic effects. It is suggested that the blockade of GSK-3 confers an indirect intercellular regulation. However, the IL-10-producing cells required for hepatic inflammatory resolution need further investigation. Targeting GSK-3 as a therapeutic strategy against liver injury is therefore suggested.

Upon TLR stimuli, regulation of IL-10 production is generally critical for immune resolution [30]. Tight regulation of GSK-3-mediated IL-10 generation has been previously reported since a critical transcriptional factor cAMP-response element-binding protein (CREB) required for IL-10 gene transactivation is suggested for use in GSK-3 regulation [38]. CREB is deactivated by active GSK-3 at the acute phase of TLR-mediated inflammatory responses. Therefore, suppressing IL-10 production is necessary for early activation of proinflammation, while active GSK-3 is also vital to sustaining TLR-induced NF-κB activation. Therefore, targeting GSK-3 could be anti-inflammatory directly by interfering with NF-κB-regulated inflammatory factor expression and indirectly causing CREB-mediated IL-10 induction. The IL-10-regulating effects raised by the blockade of GSK-3 have been widely shown in the models of liver protection [36,39]. Similar to the GSK-3 blockade, the exogenous administration and expression of IL-10 are protective in acute liver injury, including allograft liver transplantation [40], liver fibrosis [41], and immune hepatitis [42].

In a murine acute liver injury model induced by LPS and D-galactosamine (D-GalN), administrating the blocker of ER stress, 4-phenylbutyric acid, effectively rescues mice from hepatic injury and inflammation [43]. Upon ER stress, GSK-3 is activated for mediating cellular activation toward proinflammatory and proapoptotic responses [23,44]. It has been demonstrated that the blockade of ER stress also inhibits GSK-3 activation and GSK-3-mediated cell death and inflammatory activation. In brief, the inhibition of GSK-3 also confers protection from LPS- [24,45] and cecal ligation and puncture-induced liver injury [46], hemorrhagic shock [47], liver ischemia-reperfusion [36,48], and LPS/D-GalN-induced acute hepatic injury [49]. For anti-inflammation, inhibiting GSK-3 promotes autophagy to increase the expression of peroxisome proliferator-activated receptor (PPAR) α [49]. Additionally, active GSK-3 mediates ER stress to facilitate LPS-triggered hepatic inflammation [43]. Additional data have shown that in the same acute hepatic injury, the blockade of GSK-3 reduces ER stress-triggered [44] and oxidative stress-induced [50] apoptosis in hepatocytes. In studies of supplementation, including methane-rich saline [39], suberoylanilide hydroxamic acid [51], curcumin [52], and l-carnitine [53], on liver protection, all of the treatments inhibit several models of acute hepatic injury by suppressing inflammation as well as hepatocyte apoptosis. Notably, targeting GSK-3 signaling pathways for anti-inflammation and anti-apoptosis are the main effects of these liver-associated protective agents.

In addition to modulating hepatic inflammation and hepatic cell death, pharmacologically inhibiting GSK-3 by using lithium in patients with chronic hepatitis C confers antioxidant responses to avoid the progression of hepatic injury [54]. As shown in liver biopsy specimens from these patients with GSK-3 inhibition, an inactive phosphorylated GSK-3 is significantly increased and positively correlated with antioxidant Nrf2 expression. Nrf2 acts as a significant suppressor of cellular oxidative responsive pathways in the hepatic cells [55]. In saturated free fatty acid-induced hepatocyte lipoapoptosis, palmitate treatment causes GSK-3 activation, while pharmacologically inhibiting GSK-3 significantly reduced palmitate-mediated lipoapoptosis in an experimental cell culture model of Huh-7 cells. The short hairpin RNA technique to knock down GSK-3 showed that GSK-3 facilitates palmitate-induced JNK activation followed by the induction of the proapoptotic effector p53-upregulated modulator of apoptosis (PUMA) [56]. The potential treatment by targeting GSK-3 in experimental models of hepatic injury is summarized in Table 1.

## 4. Generation of IFN-γ and Its Multiple Proinflammatory Roles

IFN-γ is primarily produced by T cells, natural killer (NK) cells, and NKT cells [57,58]. Previous studies proved that the T-box transcription factor Tbx21 (T-bet) is required for IFN-γ production [59,60,61,62]. In Th1 differentiation, IFN-γ-signal transducer and activator of transcription (STAT) 1 signaling activates T-bet and then sustains the positive feedback loop to produce more IFN-γ [59,63]. T-bet may also be important in many kinds of immune cells, including CD8^+^ T cells [61,64], dendritic cells [65], B cells [66], NK cells, and NKT cells [62,67]. In general, NK and NKT cells express IFN-γ in response to infection [61,68]. Therefore, NK- and NKT-driven IFN-γ production plays a proinflammatory role in the immune hepatitis model [69]. However, the regulation of IFN-γ production by T-bet is still unclear. Following T-bet activation, T-bet (Ser^508^), which is phosphorylated by casein kinase I and GSK-3, is required for controlling cytokine production in developing Th1 cells [70].

IFN-γ generally and positively affects the production of the proinflammatory cytokine TNF-α and chemokines, including IFN-inducible protein-10, monocyte chemoattractant protein-1, monokine induced by IFN-γ, macrophage inflammatory protein-1α/β, and RANTES [58], but decreases the expression of the anti-inflammatory cytokine IL-10 [57]. In addition, IFN-γ synergizes with LPS-stimulated iNOS/NO biosynthesis [71]. Furthermore, it has been reported that IFN-γ may trigger the full activation of a variety of signaling factors, including NF-κB [72], MAPK [73], STAT1 [71], and interferon regulatory factor-1 (IRF-1) [74], to modulate its proinflammatory activation. In addition, IFN-γ induces immune cell chemotaxis into sites of inflammation through the upregulation of adhesion molecules, including intercellular adhesion molecule-1 and vascular cell adhesion molecule-1, and chemokines [75]. In brief, IFN-γ is a potent cytokine that promotes antigen processing and presentation, microbial killing, and proinflammatory cytokine production [58,68].

## 5. IFN-γ Signaling and Its Regulation

IFN-γ receptor (IFNGR) is composed of IFNGR1 and IFNGR2, which bind to Janus kinase (Jak) 1 and Jak2, respectively [58,76]. Following IFN-γ stimulation, Jak2 is autophosphorylated and activated to cause Jak1 transphosphorylation. Through Jak1-mediated IFNGR1 phosphorylation, activated IFNGR1 creates a docking site for STAT1 recruitment, followed by Jak2-mediated phosphorylation at a tyrosine residue (Tyr^701^) [58,76]. Furthermore, IFN-γ-activated MAPKs, such as ERK and p38 MAPK, subsequently phosphorylate Ser^727^ of STAT1 (Tyr^701^) to facilitate its dimerization, nuclear translocation, and DNA binding stability [77]. Beurel and Jope [78] further demonstrated the requirement of GSK-3β in facilitating IFN-γ-activated STAT3 and STAT5. This finding suggests a novel role of GSK-3β in IFN-γ signaling, but the complete regulation of GSK-3 in IFN-γ signaling remains unclear.

Critical signal components, including Jak1, Jak2, and IFNGR1, are rapidly phosphorylated within one minute of IFN-γ treatment in HeLa cells [79]. The time required for full IFN-γ-induced STAT1-IRF-1 activation and nuclear translocation is approximately thirty minutes [80]. Notably, STAT1 activation is then inhibited within one hour of IFN-γ treatment [80], and three families of proteins, SH2-containing phosphatase (SHP) 2, protein inhibitors of activated STATs, and suppressor of cytokine signaling (SOCS), have been reported to show negative inhibition of IFN-γ signaling [81,82]. SOCS proteins, including SOCS1–SOCS7, are identified as inducible negative regulators of cytokine signaling. SOCS proteins contain an SH2 domain and a carboxy-terminal SOCS box [83]. It is now known that Jak-STAT-induced SOCS1 and SOCS3 proteins subsequently interfere with Jak by repressing its activity after ligand binding [83,84]. In addition to SOCSs, dual phosphatase SHP2 can cause the dephosphorylation of Jak1, Jak2, IFNGR1, and STAT1 [85]. SHP2 becomes phosphorylated at Tyr^542^ and Tyr^580^ residues in response to growth factor stimulation [86]. However, the in-depth molecular mechanisms of SHP2 activation remain largely unclear.

## 6. GSK-3 Is Involved in IFN-γ Signaling Pathways

Targeting GSK-3 expression and activity suppresses TLR-mediated inflammation but increases anti-inflammatory cytokine IL-10 production [29,30,36]. Active GSK-3β negatively regulates the IL-10-regulating transcription factor cyclic AMP responsive element binding protein [29,87]. With a dysregulation of GSK-3-mediated excessive proinflammatory cytokine production and IL-10 downregulation, cirrhotic patients show a high risk of developing sepsis under endotoxin exposure [88]. While GSK-3 regulates the expression of NO, iNOS, and RANTES in LPS-activated macrophages, pharmacologically inhibiting GSK-3 increases IL-10 production to relieve anti-inflammation [31,88]. Accordingly, treatment with GSK-3 inhibitors comprehensively improves the survival of endotoxemic C3H/HeN mice. An advanced study demonstrated that IFN-γ treatment synergizes with TLR2-mediated IκB degradation and NF-κB activation, while TNF-α production is effectively induced by suppressing IL-10-dependent phosphorylation of STAT3 in a GSK-3-regulated manner [87,89]. In GSK-3β-deficient fetal liver cells, IFN-γ increases GSK-3β activity to reduce IL-10 expression in TLR2-stimulated cells [90]. This finding suggests that GSK-3β plays a decisive signaling role in transducing the proinflammatory activity of IFN-γ.

**Figure 3:**
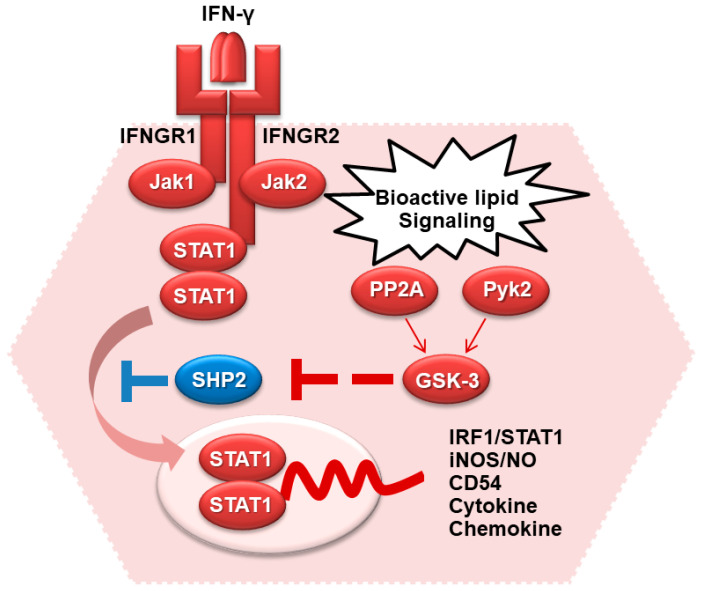
The involvement of GSK-3 in IFN-γ signaling.

Following the generation of bioactive lipid signaling, treatment of IFN-γ activates phosphatidylcholine-specific phospholipase C and PKC to cause Pyk2- and PP2A-regulated GSK-3 activation [91]. Inhibiting GSK-3 activates SHP2 to prevent STAT1 activation. Among the signaling pathways, a calcium-dependent tyrosine kinase, Pyk2, causes GSK-3β phosphorylation (Tyr^216^) and activation [34,38,92]. The involvement of GSK-3β in facilitating IFN-γ signaling has been widely investigated [34,78,87,89]; however, the mechanisms for IFN-γ-regulated GSK-3β activation remain undecided. Pyk2 can act as a downstream kinase of immunoreceptor tyrosine-based activation motif-associated receptors and causes the regulation of the IFN-induced activation of Jak-STAT [38]. Therefore, Pyk2 is involved in the regulation of Jak-STAT signaling. Moreover, Pyk2 is constitutively bound to Jak2 and undergoes tyrosine phosphorylation and activation caused by IFN-γ [93]. In response to IFN-γ-induced iNOS/NO biosynthesis, diacylglycerol is generated to activate PKC. The activations of PKC-mediated Src, Pyk2, and GSK-3β are essential for regulating IFN-γ signaling [91,94]. Importantly, our previous work [91] demonstrated the possible inhibitory effects of GSK-3 on SHP2 activation, an inhibitor of STAT1 signaling. The possible regulation of GSK-3 in facilitating IFN-γ-activated STAT1 signaling and bioactivity is summarized in Figure 3.

## 7. Immune Hepatitis

Immune-mediated hepatic injury, also called immune hepatitis, is caused by many agents, such as infectious pathogens and chemical and metal drugs [95]. Following stimulation, the condition is further induced by adverse hepatic immune responses, including activating local and infiltrated immune cells, resulting in hepatocytes undergoing apoptosis [96]. In addition, in the liver, T, NK, and NKT cells, sinusoid endothelial cells, Kupffer cells, and stellate cells are involved in hepatic immunity [97]. Therefore, advances in understanding hepatic immunopathogenesis will improve the treatment of immune hepatitis.

Many viral infections can cause chronic diseases in the liver. To mimic acute immune hepatitis, lymphocyte mitogen concanavalin A (ConA)-induced immune hepatitis closely resembles the pathology of viral-, drug-, and autoimmune-induced immune hepatitis [98]. Intravenous injection of ConA can induce immune cell infiltration in the liver and can elevate the serum alanine aminotransferase and serum aspartate aminotransferase level, followed by hepatocyte death [98]. Activated immune cells, such as T, NK, NKT, and Kupffer cells, may exhibit direct cytotoxicity or may release procytotoxic and proinflammatory cytokines to mediate liver damage [99]. NKT cells, which express invariant T-cell receptors, are an abundant cell population in the liver and play a pathogenic role in immune responses in ConA-induced immune-mediated hepatic injury [100]. In general, activated NKT cell-mediated excessive inflammatory responses may cause hepatocellular apoptosis. It has been shown that liver injury in this model depends on IFN-γ and TNF-α overproduction since administering neutralizing antibodies that recognize either cytokine effectively protects against ConA-induced immune hepatitis [101,102].

Hepatocellular apoptosis is the primary cause of hepatic injury [95]. Hepatocyte apoptosis is caused by excessive inflammation resulting from activated T cells, NKT cells, polymorphonuclear granulocytes (PMNs), and cytokine responses [96,103]. Additionally, it has been reported that ConA-induced immune hepatitis is fully protected by using macrophage depletion, T-cell depletion, and T-cell-deficient mice [98]. NKT cells increase the production of proinflammatory cytokines and procytotoxic factors, leading to hepatic injury [100,104,105,106]. Further studies showed the suppression of ConA-induced immune hepatitis in CD4^+^ neutralized mice, while the CD8^+^ neutralized mice showed no significant change [107]. PMNs are also reported to modulate the generation of IFN-γ in ConA-induced hepatic injury [103,108]. Kupffer cells are resident hepatic macrophages and can facilitate neutrophil infiltration. In Kupffer cell-depleted mice, hepatic cell apoptosis and inflammatory responses in ConA-induced immune hepatitis are reduced [109]. Upon ConA stimulation, a variety of hepatic immune cells are involved in the pathogenesis of immune hepatitis.

Several cytokine- and apoptosis-related effector molecules, including IFN-γ [101,106,110], CD95 Ligand (CD95L) [111], TNF-α [102,112], and IL-4 [104], take part in ConA-induced T cell- or NKT-mediated hepatic injury [96]. T cells are generally activated, followed by the immediate secretion of IFN-γ and TNF-α, causing cellular activation and cytotoxicity in ConA-induced hepatic injury [101]. IFN-γ-deficient mice show significant resistance to ConA-induced hepatocyte apoptosis, suggesting the proapoptotic role of IFN-γ in immune-mediated hepatic injury [113]. Hepatocytes, sinusoidal endothelial cells, stellate cells, and Kupffer cells express CD95 [114], and CD95L is generally expressed on cytotoxic T cells, NK cells, NKT cells, and hepatic macrophages [115]. Notably, the induction of CD95 expression on hepatocytes and CD95L expression on cytotoxic NKT cells after treatment with ConA is mediated by IFN-γ, and this elevated expression of CD95 causes apoptosis [113]. Furthermore, IFN-γ signaling determines the induction of multiple chemokines and adhesion molecules in ConA-induced immune hepatitis [69]. The pathogenesis of ConA-induced immune hepatitis is generally regulated by T cells, NKT cells, PMNs, cytokines, chemokines, adhesion molecules, and apoptosis.

## 8. GSK-3 in IFN-γ-Mediated Hepatic Immune Hepatitis and Its Therapeutic Efficacy

Active GSK-3 facilitates the signal transduction of IFN-γ to modulate IFN-γ-induced proinflammatory responses [34,78,87,89]. Pharmacological inhibition of GSK-3 provides anti-inflammation and cytoprotection against IFN-γ- [34,91,116], LPS- [29,31,117], and TNF-α-induced inflammation in vitro [27] and endotoxemic multiple organ failure in vivo [24,32,117,118]. In addition, the blockade of GSK-3 also has a protective effect in several IFN-γ-related autoimmune mouse models, including experimental autoimmune encephalomyelitis [25], experimental colitis [26], and type II collagen-induced arthritis [28]. Evidence has shown that IFN-γ-deficient and STAT1 mice are resistant to ConA-induced immune hepatitis [60,106,113]. It is speculated that IFN-γ-activated Jak-STAT signaling is required for ConA-induced immune hepatitis by increasing CD95/CD95L-mediated apoptosis, and GSK-3 is essential in ConA-induced IFN-γ-mediated immune hepatitis by modulating IFN-γ signaling. Previous work [106] showed that exogenous administration of ConA caused GSK-3 activation in NKT cells and hepatocytes in an in vitro cell culture model and an in vivo model of experimental immune hepatitis. The activation of GSK-3 in these cells is speculated to be important in controlling the downstream signaling of ConA-activated hepatic NKT cells as well as IFN-γ-activated hepatocytes. In the ConA-treated liver, the loss of glycogen could be observed to be accompanied by the decrease in glycogen synthase and the increase in active GSK-3 in the hepatocytes. As shown by the blockade of GSK-3 using selective inhibitors of GSK-3, the loss of glycogen is restored. While a ConA-induced liver injury is an appropriate model of glycogen deregulated disorder, our other results demonstrate that GSK-3 causes dual effects on T-bet-dependent IFN-γ production in hepatic NKT cells and IFN-γ-activated Jak2/STAT1 for proinflammatory as well as procytotoxic effects in hepatocytes. The downstream effects of GSK-3 activation are necessary for promoting IFN-γ-mediated ConA-induced immune hepatitis.

There are multiple causes of hepatic cell apoptosis in immune hepatitis. Hepatocyte apoptosis may be caused by mechanisms other than those mediated by the CD95-CD95L system because *lpr/lpr* mice showed only partial resistance against ConA-hepatitis [113,119]. Indeed, other results have shown IFN-γ-induced CD95-independent apoptosis of mouse hepatocytes in vitro [120]. Interestingly, stimulating IFN-γ effectively triggers primary hepatocyte apoptosis, probably in an IRF-1-dependent manner [121,122]. Additionally, IFN-γ-induced iNOS, a potent inducer of apoptosis [123,124], is known to be induced by IFN-γ. LPS/D-GalN-induced hepatocyte apoptosis is mediated by iNOS/NO biosynthesis [125]. IFN-γ synergizes with LPS [34] or TLR2 [87] to increase iNOS/NO biosynthesis by involving GSK-3 activation followed by inhibiting IL-10. The requirement of GSK-3 is indispensable in IFN-γ-induced iNOS expression in primary hepatocytes or Huh7 cells. Therefore, GSK-3 contributes to ConA/IFN-γ-induced iNOS/NO-mediated hepatocyte apoptosis.

The roles of GSK-3 in regulating bioactivities are diverse depending on its protein expression, activation, intracellular location, interacting molecules, and cell types [1,2,8]. This review shows the benefits of GSK-3 blockade in many acute and chronic liver diseases; however, GSK-3 may also protect hepatocytes from TNF-α-induced hepatocyte apoptosis [126]. Initially and importantly, GSK-3β deficiency causes embryonic lethality in mice since GSK-3 is required for TNF-α-activated p65 phosphorylation and upregulation of NF-κB transactivation [5]. Furthermore, during the stage of liver generation in the embryo, TNF-α-activated NF-κB is essential for hepatocyte survival by upregulating antiapoptotic protein expression [5,126] as well as iNOS/NO biosynthesis [127]. According to these findings, it is controversial in GSK-3-involved liver diseases whether targeting GSK-3 may be protective or pathogenic [10].

Furthermore, studies have shown the potential implications of inhibiting GSK-3 against septic shock and multiorgan failure [9,118]. Patients with liver cirrhosis have a high risk of developing sepsis due to excessive inflammation resulting from the deregulation of GSK-3-modulated inflammation and anti-inflammation [88]. Therefore, GSK-3 is an attractive therapeutic target of pharmacologic intervention that has become indispensable for investigation, particularly in acute liver diseases [10]. To stretch the blockade of GSK-3, inhibitors of GSK-3 are approached by using metal ions (such as lithium), which are used to block the enzymatic activity. Additionally, GSK-3 inhibitors are developed by three main classes, including ATP-competitive (such as BIO, SB216763, and SB415286), non-ATP-competitive (such as TDZD-8), and substrate competitive (such as L803) [117,128]. Additionally, modulating the upstream signaling pathways of GSK-3 activation and inactivation are suggested to be functionally regulated for controlling GSK-3. The selectivity of GSK-3 inhibitors used to suppress its intracellular activation is therefore crucial for further investigation.

## 9. Conclusions

**Figure 4:**
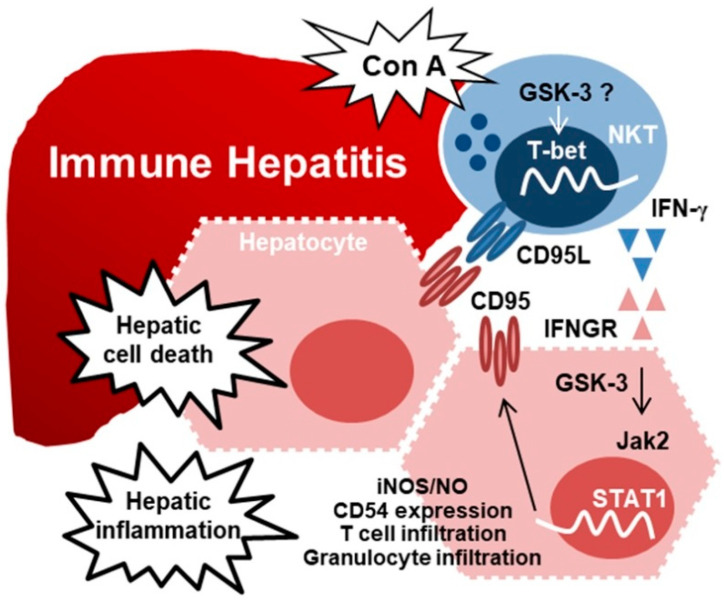
A hypothetical model for GSK-3-facilitated IFN-γ immune hepatitis. Treatment of ConA causes immune hepatitis through a mechanism involving NKT activation, hepatic cell apoptosis, and inflammatory activation. In activated NKT cells, in addition to CD95L induction, ConA induces GSK-3 activation to facilitate T-bet-modulated IFN-γ generation. Furthermore, signaling of IFN-γ and its receptor IFNGR may cause GSK-3-regulated Jak2/STAT1 signaling in hepatocytes to facilitate IFN-γ-activated Jak2-STAT1 signaling. IFN-γ is essential for inducing hepatic injury, including CD95-mediated hepatic cell death and hepatic inflammatory responses such as iNOS/NO biosynthesis, CD54 induction, and immune T cell and granulocyte infiltration. These findings illustrate a pathogenic role of GSK-3 in guiding ConA-induced immune hepatitis by facilitating IFN-γ expression, signaling, hepatic injury, and inflammation.

In summary (Figure 4), in an experimental model of ConA-induced immune hepatitis [106], activating GSK-3 by ConA determines IFN-γ generation in NKT cells and synergistically facilitates IFN-γ-activated Jak-STAT, inflammatory responses (such as CD54 expression, iNOS/NO biosynthesis, and immune cell infiltration), and proapoptotic effects (such as CD95L/CD95 signaling) in the liver, particularly in hepatocytes. GSK-3 inhibition has been used to prevent inflammatory disorders, including neurodegenerative disorders, infectious pathogens, endotoxemia, trauma, and asthma [128,129,130]. Therefore, GSK-3 inhibition represents a potential therapeutic strategy to prevent or reduce disease progression, probably through anti-inflammation and anti-apoptosis. Based on the essential roles of GSK-3 in immune hepatitis and IFN-γ signaling, drug targeting of GSK-3 and its upstream or downstream signaling can provide strategies for anti-inflammation and anti-apoptosis in immune-mediated hepatic injury.

## Figures and Tables

**Table 1:** GSK-3 in liver diseases and hepatic cell injury.

Hepatic Injury Model	The Blockade of GSK-3	References
Zymosan	4-Benzyl-2-methyl-1,2,4-thiadiazolidine-3,5-dione (TDZD-8)	[35]
IRI	SB216763/TDZD-8/Carbon monoxide	[36,37,48]
Carbon tetrachloride	Methane	[39]
LPS/D-GalN	4-Phenylbutyric acid/SB216763	[43,44,49,50]
LPS	Lithium chloride (LiCl)	[45]
CLP	SB216763	[46]
Hemorrhagic shock	TDZD-8	[47]
Transplantation	Suberoylanilide hydroxamic acid	[51]
Lead	Curcumin/l-carnitine	[52,53]
HCV	LiCl	[54]
Palmitate	GSK-3 inhibitor IX/Enzastaurin	[56]

## Data Availability

The data presented in this manuscript are available from the corresponding author upon reasonable request.

## Abbreviations

ConA	concanavalin A
CREB	cAMP-response element-binding protein
D-GalN	D-galactosamine
ER	endoplasmic reticulum
ERK	extracellular signal-regulated kinase
GSK	glycogen synthase kinase
IFN	interferon
IFNGR	IFN-γ receptor
IL	interleukin
iNOS	inducible NO synthase
IRF	interferon regulatory factor
IRI	ischemia/reperfusion injury
LiCl	lithium chloride
LPS	lipopolysaccharide
MAPK	mitogen-activated protein kinase
NF-κB	nuclear factor κB
NK	natural killer
NO	nitric oxide
PI3K	phosphatidylinositol 3-kinase
PK	protein kinase
PMNs	polymorphonuclear granulocytes
PP	protein phosphatase
Pyk	proline-rich tyrosine kinase
RANTES	regulated on activation, normal T-cell expressed and secreted
SHP	SH2-containing phosphatase
SOCS	suppressor of cytokine signaling
STAT	signal transducer and activator of transcription
T-bet	T-box transcription factor Tbx21
TDZD-8	4-Benzyl-2-methyl-1,2,4-thiadiazolidine-3,5-dione
TLR	Toll-like receptor
TNF	tumor necrosis factor
